# Nasal Cytology in Precision Rhinology: From Local Cellular Phenotyping to Personalized Management of Nasal Inflammatory Diseases

**DOI:** 10.3390/jpm16090477

**Published:** 2026-09-16

**Authors:** Matteo Gelardi

**Affiliations:** Unit of Otolaryngology, Department of Clinical and Experimental Medicine, University of Foggia, 71122 Foggia, Italy; matteo.gelardi@unifg.it

**Keywords:** nasal cytology, precision rhinology, cellular phenotyping, rhinitis, chronic rhinosinusitis, eosinophils, mast cells, neutrophils, biofilm, precision medicine

## Abstract

Nasal cytology provides a direct and minimally invasive assessment of the cellular environment of the nasal mucosa and may represent a valuable tool within the emerging framework of precision rhinology. Beyond its traditional diagnostic role, cytological examination can identify distinct local inflammatory patterns characterized by neutrophils, eosinophils, mast cells, and mixed cellular profiles, thereby revealing biological heterogeneity that may not be fully captured by clinical phenotype or systemic biomarkers alone. This review discusses the potential role of nasal cytology as a local cellular phenotyping approach in inflammatory diseases of the upper airways. Particular emphasis is placed on the interpretation of major cytological patterns, their relationship with underlying inflammatory mechanisms, and their possible integration with clinical, laboratory, and endoscopic information. The repeatability of nasal sampling also allows for longitudinal evaluation of cellular changes over time, supporting the concept of cytology as a dynamic local biomarker for monitoring disease evolution and treatment response. Nasal cytology has also recently undergone Delphi-based standardization, providing consensus recommendations that support methodological consistency, reproducibility, and broader clinical implementation. Representative original micrographs from a long-standing educational nasal cytology archive are included to illustrate the principal cellular phenotypes discussed. Although further standardization, validation, and integration with molecular biomarkers are needed, nasal cytology may contribute to a more individualized assessment of nasal inflammatory diseases by providing direct information from the target tissue. Its incorporation into multimodal diagnostic pathways could help bridge traditional rhinological evaluation and personalized management.

## 1. Introduction

Inflammatory diseases of the nasal mucosa encompass a heterogeneous group of conditions in which similar clinical manifestations may arise from markedly different underlying inflammatory mechanisms. Allergic rhinitis, non-allergic rhinitis, and chronic rhinosinusitis may therefore share symptoms such as nasal obstruction, rhinorrhea, sneezing, or olfactory impairment while differing substantially in their local cellular and molecular profiles. This biological heterogeneity has progressively supported the transition from symptom-based classification toward precision-medicine approaches based on phenotypes, endotypes, biomarkers, and treatable traits [1,2].

Precision medicine aims to identify biologically meaningful characteristics that may refine disease classification, improve prognostic assessment, and support more individualized management. In rhinology, this approach has increasingly focused on inflammatory endotypes and biomarkers, particularly in the context of type 2 inflammation [1,2]. However, systemic biomarkers do not necessarily reproduce the inflammatory environment of the nasal mucosa, and direct assessment of the target tissue may provide complementary biological information.

Nasal cytology offers a simple, minimally invasive, repeatable, and point-of-care method for directly assessing epithelial and inflammatory cells of the nasal mucosa [3]. By identifying eosinophils, mast cells, neutrophils, lymphocytes, epithelial cells, and their relative distribution, cytological examination can characterize local inflammatory patterns that may otherwise remain unrecognized during conventional clinical assessment [3]. Its clinical methodology and diagnostic applications have been progressively defined, and a recent expert-based Delphi consensus has further standardized sampling, staining, microscopic evaluation, and interpretation, providing a methodological framework for its use in both clinical practice and research [4].

Importantly, nasal cytology should not be regarded merely as a technique for detecting eosinophilia. The coexistence or predominance of different inflammatory cell populations may reveal distinct cellular phenotypes within apparently similar clinical conditions. This is particularly relevant in rhinitis, where eosinophilic, neutrophilic, mast-cell-associated, and mixed inflammatory patterns may occur across clinically overlapping conditions. Independent studies have also supported the diagnostic information provided by nasal cellular assessment, including its potential role in the evaluation of local allergic rhinitis [5] and in characterizing inflammatory cellular changes in allergic and non-allergic rhinitis [6].

Within this framework, nasal cytology may be considered a form of local cellular phenotyping capable of complementing clinical findings, allergy assessment, endoscopy, systemic biomarkers, and, when available, molecular investigations. Moreover, because nasal sampling can be repeated over time, cytology offers the possibility of monitoring changes in the local inflammatory environment during the natural course of disease or following treatment [3,4].

This review examines the role of nasal cytology within the emerging concept of precision rhinology, focusing on the principal inflammatory cellular phenotypes, their potential biological and clinical significance, and their integration into a multimodal approach to nasal inflammatory diseases. Particular attention is given to the distinction between cellular phenotype and molecular endotype, to the potential value of longitudinal cytological assessment, and to the methodological and scientific limitations that must be addressed before nasal cytology can be fully integrated into personalized management strategies.

## 2. From Nasal Cytology to Local Cellular Phenotyping

The diagnostic value of nasal cytology extends beyond the simple detection of inflammatory cells. Its major contribution lies in the possibility of recognizing reproducible cellular patterns within the nasal mucosa and relating them to different inflammatory conditions. In this perspective, the rhinocytogram provides a direct representation of the local cellular environment and may help distinguish patients who share similar symptoms but differ substantially in the composition of their inflammatory infiltrate [3,4].

This concept is particularly relevant in non-allergic rhinitis (NAR), a heterogeneous group of disorders traditionally defined largely by the absence of systemic allergic sensitization. Cytological assessment has contributed to a more detailed characterization of NAR by identifying different inflammatory patterns according to the predominant cellular population. These include non-allergic rhinitis with neutrophils (NARNE), non-allergic rhinitis with eosinophils (NARES), non-allergic rhinitis with mast cells (NARMA), and non-allergic rhinitis with eosinophils and mast cells (NARESMA) [7]. Among these patterns, NARESMA was originally characterized as a distinct and clinically more severe form associated with the simultaneous presence of eosinophilic and mast-cell inflammation [7].

The clinical relevance of this cellular heterogeneity is supported by independent observations showing that nasal eosinophilia alone cannot adequately describe the complexity of chronic rhinitis. Assessment of other inflammatory and epithelial cell populations may provide additional information for differential diagnosis and patient characterization [8]. Similarly, the usefulness of nasal cytology in distinguishing cellular forms of rhinitis has been recognized in both adult and pediatric populations [8,9].

From a precision-medicine perspective, these cytological patterns should be regarded primarily as local cellular phenotypes rather than as molecular endotypes. A cytological phenotype describes the cellular composition observed directly within the nasal mucosa, whereas an endotype implies a disease subtype defined by a specific biological or molecular mechanism. Although particular cell populations may suggest the predominance of specific inflammatory pathways, cytological findings alone cannot establish the underlying molecular mechanism. This distinction is essential to avoid overinterpretation and to place nasal cytology correctly within an integrated diagnostic framework.

Nevertheless, the direct visualization of local inflammatory cells provides information that may complement clinical phenotype, systemic biomarkers, and allergy testing. In this context, nasal cytology may contribute to precision-oriented diagnostic strategies by providing direct characterization of the local inflammatory cellular profile [10,11].

Thus, the transition from descriptive rhinocytology to local cellular phenotyping represents a conceptual evolution of nasal cytology. Rather than assigning a single diagnosis on the basis of one cellular finding, the objective becomes the recognition of the dominant and coexisting cellular components of inflammation and their integration with the broader clinical and biological profile of the individual patient.

## 3. Major Cytological Patterns in Nasal Inflammatory Diseases

**Figure 1:**
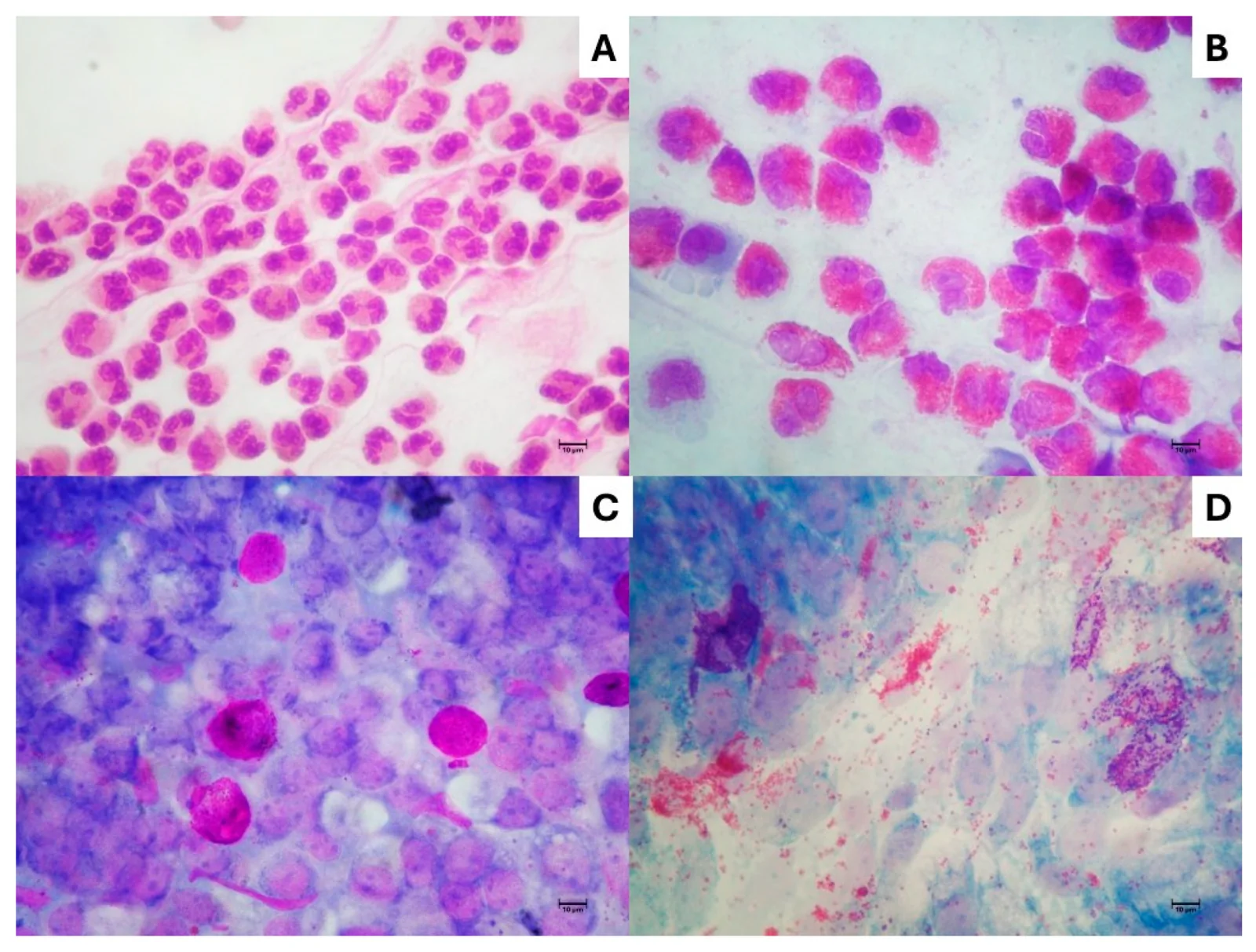
**Representative cytological patterns in nasal inflammatory diseases.** (**A**) Neutrophilic pattern. (**B**) Eosinophilic pattern. (**C**) Mast-cell-associated pattern. (**D**) Mixed eosinophilic/mast-cell pattern (NARESMA), characterized by marked degranulation of both eosinophils and mast cells. May–Grünwald–Giemsa staining. Scale bar = 10 μm.

Nasal inflammatory diseases are characterized by marked cellular heterogeneity. Although clinical manifestations may overlap considerably, cytological examination of the nasal mucosa can reveal distinct inflammatory profiles according to the predominant or coexisting cellular populations. Recognition of these patterns represents the practical basis of local cellular phenotyping and may provide complementary information beyond conventional clinical classification [10,11]. Representative examples of the principal cytological patterns are shown in Figure 1.

These original micrographs from the author’s educational nasal cytology archive were developed over approximately 25 years of routine microscopic observation and teaching activity. The images are fully anonymized and are presented solely for educational and illustrative purposes.

### 3.1. Neutrophilic Pattern

A predominantly neutrophilic infiltrate is frequently observed in nasal cytology and may occur in several inflammatory conditions, including non-allergic rhinitis, infectious or irritative processes, and environmental exposure [8,11]. Neutrophils represent one of the principal effector cells of innate inflammation and are rapidly recruited to mucosal sites in response to tissue injury, infection, or irritative stimuli. Their persistence generally reflects ongoing inflammatory recruitment rather than a disease-specific mechanism.

Importantly, the presence of neutrophils should not automatically be interpreted as evidence of bacterial infection. Activated neutrophils release a broad spectrum of biologically active mediators, including neutrophil elastase, myeloperoxidase, reactive oxygen species, defensins, and other proteolytic enzymes. Although these mediators are essential for host defense, excessive or persistent neutrophilic activation may promote epithelial injury, mucus hypersecretion, and disruption of mucosal homeostasis. Neutrophil elastase, in particular, may stimulate mucin production and secretion, providing a biological link between intense neutrophilic inflammation and epithelial dysfunction [12].

Within the spectrum of non-allergic rhinitis, a neutrophil-predominant cytological phenotype has traditionally been described as non-allergic rhinitis with neutrophils (NARNE) [3,9]. Neutrophilic inflammation may also occur in upper-airway irritation associated with laryngopharyngeal reflux. Clinical observations suggest an association between reflux-related symptoms and nasal complaints, particularly nasal obstruction, in patients with non-allergic rhinitis [13]. Reflux-related inflammation may also involve the nasopharyngeal region and has been associated with impaired Eustachian tube function, potentially contributing in selected patients to aural fullness or other symptoms of tubal dysfunction [14].

**Figure 2:**
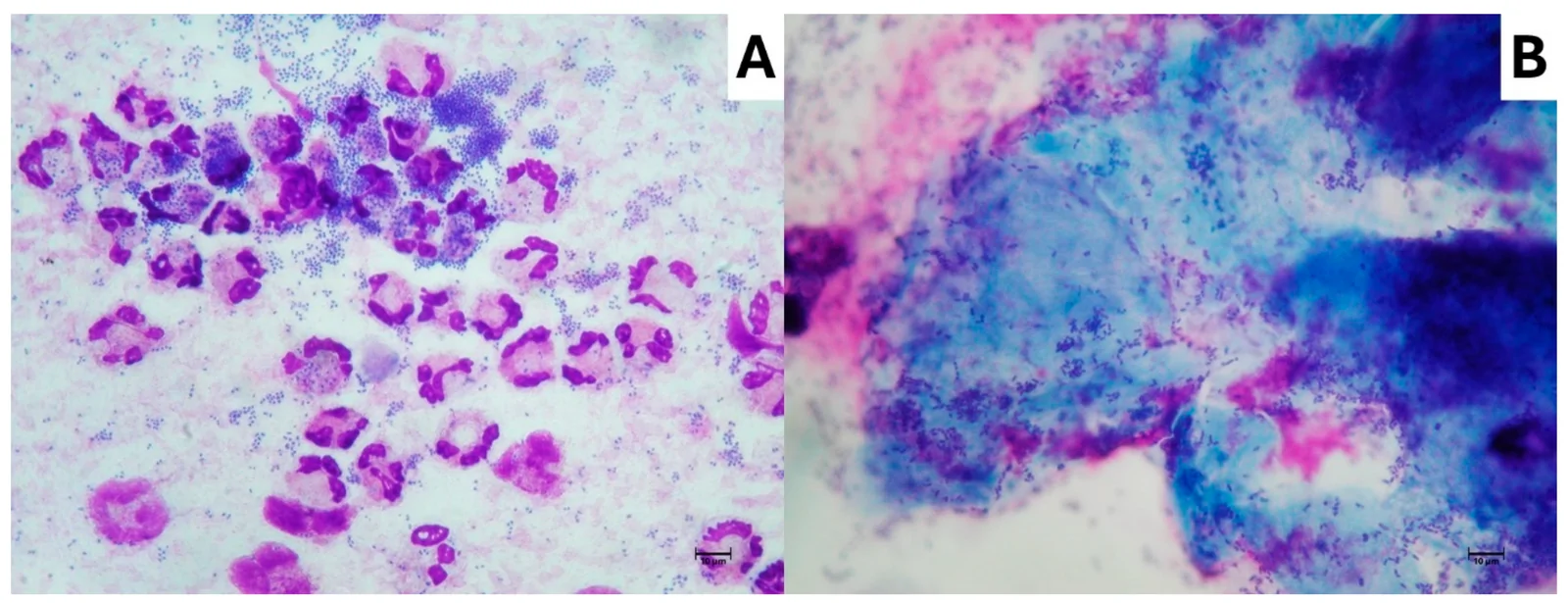
**Cytological findings in bacterial and biofilm-associated nasal inflammation.** (**A**) Bacterial rhinitis characterized by numerous neutrophils and abundant extracellular and intracellular bacteria, the latter representing a morphological expression of phagocytic activity. (**B**) Biofilm-associated pattern showing numerous bacterial aggregates embedded within an extracellular matrix. The characteristic cyan chromatic appearance corresponds to the morphological pattern historically described as an “infectious spot”. May–Grünwald–Giemsa staining. Scale bar = 10 μm. Original micrographs from the author’s educational nasal cytology archive, developed over approximately 25 years of routine microscopic observation and teaching activity. The images are fully anonymized and are presented solely for educational and illustrative purposes.

In infectious conditions, however, the neutrophilic pattern acquires particular diagnostic relevance. Bacterial rhinitis may be characterized cytologically by numerous neutrophils associated with abundant bacterial elements, both extracellular and intracellular. The presence of bacteria within neutrophils represents a morphological expression of phagocytic activity and provides direct microscopic evidence of the interaction between the host inflammatory response and microorganisms (Figure 2A).

Particularly in chronic infectious or inflammatory conditions, microorganisms may also organize into biofilm. Nasal cytology can reveal characteristic morphological–chromatic aggregates in which numerous bacteria are embedded within an extracellular matrix adherent to the mucosal surface. This pattern was originally described as the “infectious spot”, characterized by a distinctive cyan appearance at light microscopy and subsequently related to the presence of a polysaccharide-rich biofilm matrix [15] (Figure 2B).

The identification of biofilm-associated features broadens the information provided by nasal cytology beyond conventional inflammatory-cell counting. In this setting, the rhinocytogram may simultaneously document neutrophilic inflammation, microbial burden, phagocytic activity, and biofilm-associated organization, providing a morphological representation of the local host–microorganism interaction. More recent work has further emphasized the potential role of nasal cytology in the diagnostic assessment of sino-nasal biofilm-associated disease [16].

Thus, a marked neutrophilic cytological pattern should be interpreted within the overall clinical context, taking into account infectious, irritative, environmental, and reflux-related factors, as well as associated epithelial and microbial findings, rather than being regarded as a disease-specific feature.

### 3.2. Eosinophilic Pattern

Eosinophilic inflammation represents one of the most recognizable and clinically relevant cytological patterns of the nasal mucosa. It is commonly associated with allergic rhinitis, but eosinophils may also be detected in patients without demonstrable systemic allergic sensitization, as occurs in non-allergic rhinitis with eosinophilia syndrome (NARES) and in selected patients with local allergic rhinitis (LAR) [5,17].

Nasal eosinophilia has been associated with the severity of local inflammatory activity and with clinical manifestations of allergic rhinitis [17]. However, its presence is not disease-specific. Similar eosinophilic infiltrates may occur in different clinical settings, making it essential to integrate cytological findings with allergy testing, clinical history, endoscopic evaluation, and disease phenotype.

Importantly, cytological assessment may provide information beyond the mere number of eosinophils. Eosinophils may appear intact or show variable degrees of degranulation, with extracellular dispersion of cytoplasmic granules. Such findings represent morphological evidence of eosinophil activation and may provide additional information on the current inflammatory state of the nasal mucosa. Rather than being interpreted as a stand-alone marker of disease severity or as an indication for a specific treatment, eosinophil degranulation may contribute to the overall assessment of inflammatory activity and could potentially be followed during therapy.

In eosinophilic chronic rhinosinusitis, particularly CRSwNP, intense eosinophilic inflammation may also be accompanied by eosinophil-rich mucin and Charcot–Leyden crystals. These crystals, largely composed of galectin-10, are closely associated with activated and cytolytic eosinophils and represent a characteristic morphological feature of eosinophilic inflammation. Their presence in sinonasal secretions or tissue has been associated with more intense type 2 inflammatory disease and, in some studies, with an increased risk of nasal polyp recurrence [18,19].

These observations further emphasize that the eosinophilic pattern should not be reduced to an eosinophil count alone. Cell morphology, degranulation, extracellular products, eosinophilic mucin, and Charcot–Leyden crystals may together provide a richer morphological representation of local eosinophilic inflammation.

From a precision-rhinology perspective, the identification of an eosinophilic pattern therefore provides direct evidence of a local eosinophil-rich inflammatory phenotype rather than establishing a specific diagnosis or molecular endotype. Its interpretation may become increasingly informative when combined with clinical phenotype, systemic biomarkers, and longitudinal assessment of changes induced by treatment.

### 3.3. Mast-Cell-Associated Pattern

Mast cells are another important component of nasal mucosal inflammation. They may be observed in allergic disease and in selected forms of non-allergic rhinitis, including the cytological pattern historically defined as non-allergic rhinitis with mast cells (NARMA) [3,9].

Their identification by nasal cytology may provide additional information on the local inflammatory environment, particularly when conventional allergy testing does not fully explain the patient’s symptoms. Nevertheless, the presence of mast cells should not be interpreted as direct evidence of a specific molecular pathway, because cytology depicts cellular morphology and distribution rather than mediator release or molecular activation.

The mast-cell-associated pattern is therefore best considered a distinct local cellular phenotype whose clinical significance should be interpreted together with the broader inflammatory and clinical profile of the patient.

### 3.4. Mixed Eosinophilic/Mast-Cell Pattern

The coexistence of eosinophils and mast cells represents a particularly relevant example of mixed cellular inflammation. This cytological pattern has been described as non-allergic rhinitis with eosinophils and mast cells (NARESMA) and was initially associated with a more severe clinical phenotype than other cytological forms of non-allergic rhinitis [7].

The clinical relevance of eosinophil–mast-cell co-infiltration has subsequently been extended to chronic rhinosinusitis with nasal polyps (CRSwNP), where an intraepithelial eosinophil–mast-cell pattern was associated with greater disease severity. This observation supports the potential clinical and prognostic significance of mixed local cellular inflammation beyond non-allergic rhinitis [20].

Beyond the simple simultaneous presence of the two cell populations, cytological examination may also reveal clear signs of cellular activation. In some cases, eosinophils and mast cells appear partially or markedly degranulated, with extracellular dispersion of their cytoplasmic granules within the surrounding mucosal background. This finding provides a morphological indication of an activated local inflammatory environment and further emphasizes the biological complexity of the mixed eosinophilic/mast-cell phenotype.

The representative NARESMA pattern shown in Figure 1D illustrates this aspect particularly well, with both eosinophils and mast cells displaying marked degranulation. Such a cytological picture should not be interpreted as a direct surrogate of a specific molecular pathway, but rather as a morphological expression of active local inflammation that may complement clinical and laboratory information.

More broadly, the recognition of mixed inflammatory patterns challenges rigid binary classifications such as allergic versus non-allergic rhinitis. It supports a more nuanced interpretation based on the actual cellular composition and activation status of the nasal mucosa [7,10,11].

### 3.5. Cytological Patterns as a Visual Readout of Local Inflammation

The principal advantage of nasal cytology lies in its ability to make the inflammatory environment of the target tissue directly visible. Whereas systemic biomarkers provide indirect information on inflammatory activity, the rhinocytogram offers a morphological representation of the cells currently present within the nasal mucosa.

This direct cellular readout may be particularly informative in patients with similar clinical phenotypes but different underlying inflammatory profiles. Moreover, the potential clinical relevance of cytological stratification has been explored in allergic rhinitis, where treatment individualized according to nasal cytological findings has been associated with improved clinical outcomes [21].

These observations support the concept that local cellular phenotyping may contribute to a more individualized interpretation of nasal inflammatory diseases. However, cytology should be regarded as one component of a multimodal diagnostic framework rather than as an isolated treatment-guiding system, and further prospective validation remains necessary before specific cytological patterns can be linked to standardized therapeutic algorithms. The main cytological patterns and their potential relevance in precision rhinology are summarized in Table 1.

Cytological patterns represent local cellular phenotypes and should not be considered equivalent to molecular endotypes. Their interpretation requires integration with clinical, allergological, endoscopic, systemic, and, when available, molecular data.

## 4. From Cellular Phenotype to Personalized Management

The transition from conventional disease classification to precision rhinology requires the integration of multiple sources of information. Clinical phenotype, allergy assessment, endoscopic findings, systemic biomarkers, and, when available, molecular data each describe different aspects of inflammatory disease. Within this framework, nasal cytology may contribute an additional layer of information by directly characterizing the cellular composition of the target mucosa [1,2].

An example of this integrative approach is provided by Clinical-Cytological Grading (CCG) in CRSwNP, which combines the predominant cytological pattern with major clinical comorbidities to achieve a more biologically informed stratification of disease severity and recurrence risk [22]. Although CCG should not be interpreted as a stand-alone therapeutic algorithm, it illustrates how local cellular information can be incorporated into broader precision-medicine frameworks.

This distinction is clinically relevant because patients sharing the same diagnostic label may display different inflammatory profiles. Conversely, similar cellular patterns may be observed across different clinical entities. The presence of eosinophilic, neutrophilic, mast-cell-associated, or mixed inflammation should therefore not be considered sufficient to define a disease or dictate a specific treatment. Rather, cytological findings should be interpreted as one component of an integrated inflammatory profile.

The concept is consistent with the broader evolution of rhinology toward phenotype- and endotype-based management. In chronic rhinosinusitis, increasing attention has been directed toward inflammatory markers and endotypes that may improve patient stratification and support more individualized therapeutic decisions [23,24]. However, biomarkers differ in biological meaning, accessibility, reproducibility, and clinical applicability, and no single marker is currently sufficient to represent the complexity of mucosal inflammation [23].

Nasal cytology has several potential advantages within this multimodal approach. It provides direct information from the nasal mucosa, is minimally invasive, can be performed repeatedly, and allows immediate visualization of both predominant and coexisting inflammatory cell populations [3,4]. These characteristics may be particularly useful when clinical findings and systemic biomarkers are discordant or when apparently similar patients show different inflammatory behavior.

From a practical clinical perspective, nasal cytology should not necessarily be regarded as a routine investigation for every patient presenting with common nasal symptoms, nor should its use be restricted exclusively to patients who have failed empirical therapy. Its greatest value may lie in selected patients in whom local inflammatory phenotyping can refine diagnostic interpretation or contribute to individualized management. This may include persistent, recurrent, atypical, or poorly controlled disease, discordance between symptoms and conventional diagnostic findings, and clinical settings in which differentiation among eosinophilic, neutrophilic, mast-cell-associated, or mixed inflammatory patterns may provide additional relevant information.

In selected high-risk patients, local cellular phenotyping may also provide a rationale for closer interaction between rhinologists and pathologists. This may be particularly relevant in CRSwNP characterized by mixed eosinophilic–mast-cell inflammation, especially when associated with asthma and NSAID-exacerbated respiratory disease (N-ERD). In these settings, complementary histopathological assessment using mast-cell markers such as tryptase and CD117 may help characterize the mast-cell component more accurately and integrate cytological findings with tissue-level information. Such a multidisciplinary approach should not be regarded as routine for every patient, but may be valuable in selected severe or recurrent phenotypes in which a more detailed definition of the inflammatory cellular landscape could influence biological interpretation and future therapeutic stratification [20,22].

At the same time, local cellular phenotyping will need to evolve in parallel with therapeutic innovation. The expanding availability of treatments directed toward increasingly specific biological targets, including defined cell populations, cytokines, chemokines, receptors, and intracellular pathways, is progressively changing the therapeutic landscape of inflammatory airway diseases. In this context, an exclusively eosinophil-centered interpretation of nasal inflammation may become increasingly insufficient. A broader and more detailed characterization of all cellular populations present within the nasal mucosa, including their coexistence, activation state, and changes over time, may provide a more informative representation of the local inflammatory environment. The ultimate goal should not be to infer a molecular mechanism from morphology alone, but to integrate cellular, molecular, and clinical information so that a specific biological target can be interpreted within the corresponding local cellular context. Such an approach may represent one of the most tangible expressions of precision medicine in rhinology.

Evidence that cytological information may contribute to individualized treatment has already emerged in allergic rhinitis. In an independent clinical study, treatment tailored according to nasal cytological findings was associated with improved clinical outcomes compared with conventional management [21]. Although these observations are encouraging, they should not be interpreted as evidence that cytology alone can determine therapeutic choice. Prospective studies using standardized cytological criteria and predefined treatment algorithms are still required.

A pragmatic model of precision rhinology may therefore be based on the progressive integration of clinical phenotype, local cellular phenotype, systemic and local biomarkers, and disease-specific investigations. Within such a model, nasal cytology does not replace established diagnostic procedures but adds direct information on the inflammatory status of the target tissue.

Ultimately, the value of local cellular phenotyping lies not in assigning patients to rigid cytological categories, but in reducing biological uncertainty. By revealing inflammatory heterogeneity that may remain hidden behind the same clinical diagnosis, nasal cytology may contribute to more informed and individualized management of nasal inflammatory diseases.

**Figure 3:**
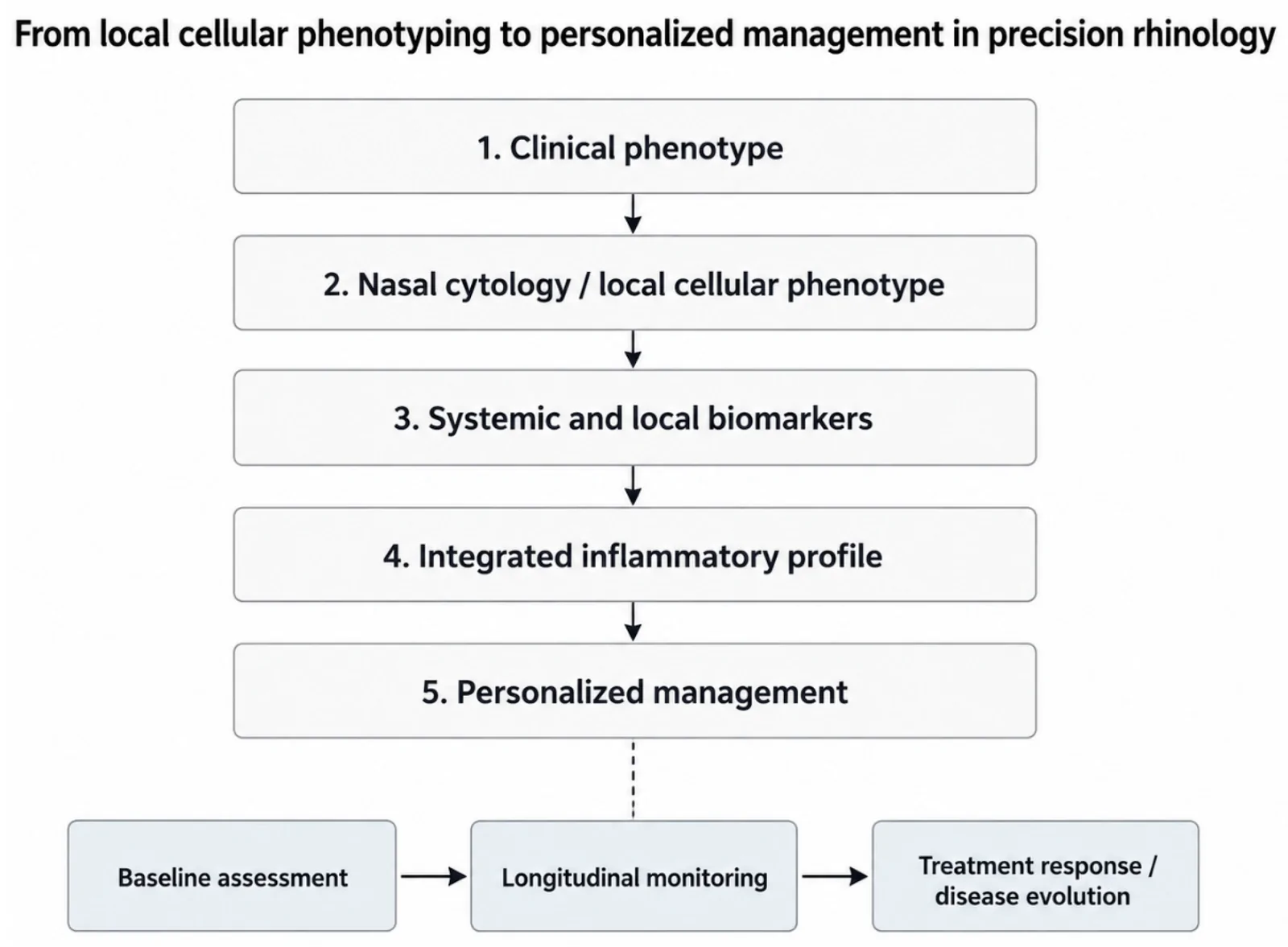
**From local cellular phenotyping to personalized management in precision rhinology.** Schematic representation of the conceptual role of nasal cytology within a multimodal precision-rhinology framework. Clinical phenotype, nasal cytology, and systemic/local biomarkers contribute to the definition of an integrated inflammatory profile, which may support more personalized management of nasal inflammatory diseases. Because nasal cytology is repeatable over time, it may also contribute to baseline assessment, longitudinal monitoring, and evaluation of treatment response or disease evolution.

This integrated framework is schematically summarized in Figure 3.

## 5. Longitudinal Cytology as a Dynamic Local Biomarker

One of the distinctive advantages of nasal cytology is its repeatability. Unlike tissue biopsy, nasal sampling can be performed serially with minimal burden to the patient, allowing the inflammatory cellular profile of the mucosa to be assessed not only at a single time point but also throughout the course of disease and treatment [3,4].

This longitudinal dimension changes the conceptual role of nasal cytology. A rhinocytogram should not necessarily be regarded as a static representation of a fixed inflammatory phenotype. Rather, cellular composition may evolve according to disease activity, environmental exposure, infections, pharmacological treatment, and biological therapy. Repeated assessment may therefore provide information on the direction and magnitude of changes occurring within the local inflammatory environment.

At present, no universally established interval has been defined for repeating nasal cytology. The timing of reassessment should therefore be individualized according to the clinical context, disease activity, and therapeutic intervention. Repeat cytology may be particularly informative when a clinically meaningful change in symptoms occurs, after initiation or modification of treatment, or during the longitudinal follow-up of persistent or recurrent disease. Thus, rather than being performed according to a rigid predefined schedule, serial rhinocytograms should be obtained when reassessment of the local inflammatory profile may provide clinically relevant information.

Evidence supporting this concept has emerged particularly in chronic rhinosinusitis with nasal polyps. Nasal cytology has been shown to provide a non-invasive assessment of local type 2 inflammation and to correlate with tissue eosinophilic involvement [25]. More importantly, differential nasal cytology performed during dupilumab treatment demonstrated a significant reduction in nasal eosinophils over time, supporting its potential use as a non-invasive method for monitoring biological changes during targeted therapy [26].

These observations introduce an important distinction between a predictive biomarker and a dynamic monitoring biomarker. A baseline cytological pattern may not necessarily predict whether an individual patient will respond to a specific treatment. Nevertheless, serial changes in cellular populations may provide direct evidence that the local inflammatory environment is being modified during therapy [26].

The potential value of longitudinal cytology extends beyond eosinophils. Future studies should investigate whether changes in mast cells, neutrophils, lymphocytes, epithelial cells, and mixed inflammatory patterns provide additional information on treatment response, persistence of inflammation, or transition between inflammatory states. Such an approach would be particularly relevant as therapeutic strategies increasingly target different components of the inflammatory network.

Therefore, the longitudinal assessment of nasal cellularity may transform nasal cytology from a diagnostic snapshot into a dynamic local biomarker. Its greatest potential may lie not in defining a patient permanently by a single cytological category, but in documenting how the local cellular environment changes over time and in response to increasingly targeted therapeutic interventions.

## 6. Limitations and Future Perspectives

Despite its potential advantages, nasal cytology still presents several methodological and interpretative limitations that should be considered before its broader incorporation into precision-rhinology pathways. Cytological findings are influenced by sampling technique, site of collection, staining quality, microscopic experience, and criteria used for cell identification and quantification. Although previous studies have shown good reproducibility under standardized conditions, variability related to sampling and observer interpretation remains an important methodological issue [27]. More recently, an expert-based Delphi consensus has provided a standardized framework for sampling, staining, microscopic evaluation, and reporting, representing an important step toward greater methodological uniformity [4].

Standardization of the sampling site and collection procedure is particularly important. Nasal cytological specimens should preferably be obtained from a consistent and representative mucosal site, typically the middle portion of the inferior turbinate, using a standardized scraping technique that minimizes traumatic contamination and preserves cellular morphology. Sampling, immediate slide preparation, staining, and microscopic evaluation should follow standardized procedures, and the same anatomical site and technique should preferably be maintained when serial examinations are compared over time. These pre-analytical aspects are essential because differences in collection site or sampling procedure may substantially influence the cellular composition observed in the rhinocytogram.

Nasal secretions, lavage fluid, or aspirated material may also contain diagnostically useful inflammatory cells and extracellular components. However, these specimens should not be regarded as fully interchangeable with standardized direct mucosal sampling. Their cellular composition may be influenced by mucus accumulation, dilution, drainage from different sinonasal compartments, and local disease activity. Accordingly, secretion-based samples may provide complementary information, whereas direct sampling from a defined mucosal site remains preferable when reproducible cellular phenotyping and longitudinal comparison are the primary objectives.

A further limitation concerns the biological interpretation of cytological findings. Nasal cytology directly describes the cellular composition of the sampled mucosal surface but does not, by itself, define the molecular pathways responsible for recruitment, activation, or persistence of those cells. Accordingly, an eosinophilic, neutrophilic, mast-cell-associated, or mixed cytological phenotype should not be considered equivalent to a molecular endotype. The same cellular population may participate in different inflammatory pathways, while apparently similar cytological patterns may conceal substantial molecular heterogeneity.

Sampling location must also be considered. Routine nasal cytology generally reflects the cellular environment of the accessible nasal mucosa and cannot necessarily be assumed to reproduce inflammation occurring within all sinonasal compartments. This limitation is particularly relevant in chronic rhinosinusitis, where inflammatory patterns may vary according to anatomical site and disease phenotype. Cytological information should therefore be interpreted as a local mucosal readout and integrated with endoscopic, radiological, clinical, and, when appropriate, tissue-based findings.

The next major step should be the integration of morphology with molecular biology. Transcriptomic and single-cell approaches are increasingly revealing a much greater diversity of epithelial, stromal, and immune-cell states within chronically inflamed sinonasal mucosa than can be captured by conventional inflammatory classifications alone. Such technologies do not diminish the value of cytology; rather, they offer the opportunity to connect readily observable cellular phenotypes with more specific molecular programs. Single-cell studies in CRSwNP have already demonstrated substantial heterogeneity among epithelial and stromal populations, reinforcing the need to move beyond simplified inflammatory categories [28].

Digital cytology and artificial intelligence may further contribute to this evolution. Automated image-analysis systems have already demonstrated the feasibility of detecting and classifying nasal cytological cells from digital microscopic images, potentially reducing observer dependence and facilitating quantitative assessment [29]. More recent whole-slide image approaches have extended this concept toward automated quantitative inflammatory phenotyping, suggesting that digital platforms could eventually support standardized cell counting, recognition of mixed inflammatory profiles, and longitudinal comparison of serial samples [30].

Future research should therefore focus not only on improving reproducibility but also on expanding the spectrum of cellular information extracted from the nasal mucosa. Particular attention should be given to neutrophils, mast cells, lymphocytes, epithelial-cell alterations, mixed inflammatory profiles, and cellular activation states, rather than maintaining an exclusively eosinophil-centered perspective. This broader approach will become increasingly relevant as therapeutic strategies target more specific cells, cytokines, chemokines, receptors, and intracellular pathways.

An additional barrier to wider implementation is educational rather than technological. Despite its methodological simplicity, nasal cytology is not yet systematically incorporated into rhinology training and residency curricula, limiting the number of clinicians with adequate experience in sampling, slide interpretation, and cellular pattern recognition. This represents an important implementation gap [3,4]. Where structured teaching programs and dedicated training networks have been developed, nasal cytology has become more readily integrated into clinical practice and has facilitated collaborative research and the development of scientific communities devoted to the field. Broader incorporation of nasal cytology into specialist education, residency programs, and continuing medical education may therefore be as important as further technological development for its future dissemination.

Ultimately, the future of nasal cytology may lie in its integration within a multimodal framework combining morphology, quantitative digital analysis, molecular biomarkers, clinical phenotype, and longitudinal assessment. Such integration could transform a relatively simple microscopic technique into a biologically richer platform for local inflammatory phenotyping while preserving its major practical advantages: accessibility, repeatability, low invasiveness, and direct visualization of the target mucosa.

## 7. Conclusions

Nasal cytology offers a direct, minimally invasive, and repeatable view of the cellular environment of the nasal mucosa. Its value extends beyond the identification of eosinophilia, allowing recognition of neutrophilic, mast-cell-associated, eosinophilic, and mixed inflammatory patterns that may add biologically relevant information to conventional clinical classification.

Within the framework of precision rhinology, the future role of nasal cytology should therefore move from an exclusively eosinophil-centered perspective toward a broader concept of comprehensive local cellular phenotyping. Such an approach may become increasingly relevant as therapeutic strategies are progressively directed toward specific biological targets, including distinct cell populations, cytokines, chemokines, receptors, and intracellular pathways.

Nasal cytology should not be considered a substitute for molecular biomarkers or established diagnostic procedures, but rather as a complementary tool capable of linking morphology, local inflammation, and longitudinal disease assessment. Its integration with clinical phenotype, systemic and local biomarkers, digital image analysis, and molecular profiling may ultimately contribute to a more individualized interpretation and management of nasal inflammatory diseases.

## Figures and Tables

**Table 1:** Main cytological patterns in nasal inflammatory diseases and their potential relevance in precision rhinology.

Cytological Pattern	Main Cytological Finding	Possible Biological Interpretation	Potential Clinical Contribution
**Neutrophilic**	Predominance of neutrophils	Local neutrophilic inflammation related to irritative, infectious, environmental, or non-allergic mechanisms	Supports characterization of non-eosinophilic inflammation; should not be interpreted as synonymous with bacterial infection
**Eosinophilic**	Predominance of eosinophils	Local eosinophil-rich inflammation, frequently associated with type 2 inflammatory pathways	Complements allergy testing and systemic biomarkers; may identify local eosinophilic inflammation even in the absence of systemic sensitization
**Mast-cell-associated**	Presence or predominance of mast cells	Local mast-cell involvement and possible cellular activation	Adds information on the local inflammatory environment not necessarily reflected by peripheral biomarkers
**Mixed eosinophilic/mast-cell (NARESMA)**	Coexistence of eosinophils and mast cells, often with degranulation	Complex and potentially more active local inflammatory phenotype	May be associated with greater disease severity and provides a broader representation of type 2 cellular inflammation
**Mixed/other inflammatory profiles**	Coexistence of multiple inflammatory cell populations	Biological heterogeneity not adequately represented by a single-cell classification	Supports comprehensive local cellular phenotyping and integration with clinical and molecular information

## Data Availability

No new datasets were generated or analyzed in this study.

## References

[1] Hellings P.W., Fokkens W.J., Bachert C., Akdis C.A., Bieber T., Agache I., Bernal-Sprekelsen M., Canonica G.W., Gevaert P., Joos G. (2017). Positioning the principles of precision medicine in care pathways for allergic rhinitis and chronic rhinosinusitis: A EUFOREA-ARIA-EPOS-AIRWAYS ICP statement. Allergy.

[2] Gurrola J., Borish L. (2017). Chronic rhinosinusitis: Endotypes, biomarkers, and treatment response. J. Allergy Clin. Immunol..

[3] Heffler E., Landi M., Caruso C., Fichera S., Gani F., Guida G., Liuzzo M.T., Pistorio M.P., Pizzimenti S., Riccio A.M. (2018). Nasal cytology: Methodology with application to clinical practice and research. Clin. Exp. Allergy.

[4] Macchi A., Gelardi M., Landi M., Gramellini G., Giancaspro R., Nappi L., Imperatore C., Bocciolini C., Seccia V., Pecoraro P. (2026). Standardization of Nasal Cytology: An Expert-based Delphi Consensus. Curr. Allergy Asthma Rep..

[5] Phothijindakul N., Chusakul S., Aeumjaturapat S., Kanjanaumporn J., Snidvongs K., Ruangritchankul K., Phannaso C. (2019). Nasal Cytology as a Diagnostic Tool for Local Allergic Rhinitis. Am. J. Rhinol. Allergy.

[6] Kovalhuk L.C.S., Telles E.Q., Lima M.N., Rosario Filho N.A. (2020). Nasal lavage cytology and mucosal histopathological alterations in patients with rhinitis. Braz. J. Otorhinolaryngol..

[7] Gelardi M., Maselli Del Giudice A., Fiorella M.L., Fiorella R., Russo C., Soleti P., Di Gioacchino M., Ciprandi G. (2008). Non-allergic rhinitis with eosinophils and mast cells constitutes a new severe nasal disorder. Int. J. Immunopathol. Pharmacol..

[8] Canakcioglu S., Tahamiler R., Saritzali G., Alimoglu Y., Isildak H., Guvenc M.G., Acar G.O., Inci E. (2009). Evaluation of nasal cytology in subjects with chronic rhinitis: A 7-year study. Am. J. Otolaryngol..

[9] Günizi H., Orhan Kubat G., Günizi Ö.C., Sevil E., Yıldırım E., Eren A. (2026). Nasal eosinophilia in pediatric non-allergic rhinitis: Correlation with clinical severity scales. BMC Pediatr..

[10] Ciofalo A., Cavaliere C., Incorvaia C., Plath M., Ridolo E., Pucciarini F., Altissimi G., Greco A., De Vincentiis M., Masieri S. (2022). Diagnostic performance of nasal cytology. Eur. Arch. Otorhinolaryngol..

[11] Myszkowska D., Bazgier M., Brońska S., Nowak K., Ożga J., Woźniak A., Stanisz A., Szaleniec J. (2022). Scraping nasal cytology in the diagnostics of rhinitis and the comorbidities. Sci. Rep..

[12] Shao M.X.G., Nadel J.A. (2005). Neutrophil elastase induces MUC5AC mucin production in human airway epithelial cells via a cascade involving protein kinase C, reactive oxygen species, and TNF-alpha-converting enzyme. J. Immunol..

[13] Hamizan A.W., Choo Y.Y., Loh P.V., Abd Talib N.F., Mohd Ramli M.F., Zahedi F.D., Husain S. (2021). The association between the reflux symptoms index and nasal symptoms among patients with non-allergic rhinitis. J. Laryngol. Otol..

[14] Brunworth J.D., Mahboubi H., Garg R., Johnson B., Brandon B., Djalilian H.R. (2014). Nasopharyngeal acid reflux and Eustachian tube dysfunction in adults. Ann. Otol. Rhinol. Laryngol..

[15] Gelardi M., Passalacqua G., Fiorella M.L., Mosca A., Quaranta N. (2011). Nasal cytology: The “infectious spot”, an expression of a morphological-chromatic biofilm. Eur. J. Clin. Microbiol. Infect. Dis..

[16] Gelardi M., Giancaspro R., Cassano M. (2023). Biofilm in sino-nasal infectious diseases: The role nasal cytology in the diagnostic work up and therapeutic implications. Eur. Arch. Otorhinolaryngol..

[17] Ciprandi G., Vizzaccaro A., Cirillo I., Tosca M.A., Massolo A., Passalacqua G. (2005). Nasal eosinophils display the best correlation with symptoms, pulmonary function and inflammation in allergic rhinitis. Int. Arch. Allergy Immunol..

[18] Ueki S., Miyabe Y., Yamamoto Y., Fukuchi M., Hirokawa M., Spencer L.A., Weller P.F. (2019). Charcot-Leyden Crystals in Eosinophilic Inflammation: Active Cytolysis Leads to Crystal Formation. Curr. Allergy Asthma Rep..

[19] Wu D., Yan B., Wang Y., Zhang L., Wang C. (2021). Predictive Significance of Charcot-Leyden Crystal Protein in Nasal Secretions in Recurrent Chronic Rhinosinusitis with Nasal Polyps. Int. Arch. Allergy Immunol..

[20] Gelardi M., Giancaspro R., Duda L., Quaranta V.N., Pizzulli C., Maiorano E., Di Canio F.M., Ruzza A., Iannuzzi L., Quaranta N.A.A. (2023). Eosinophil-mast cell pattern of intraepithelial infiltration as a marker of severity in CRSwNP. Sci. Rep..

[21] Chen J., Zhou Y., Zhang L., Wang Y., Pepper A.N., Cho S.H., Kong W. (2017). Individualized Treatment of Allergic Rhinitis According to Nasal Cytology. Allergy Asthma Immunol. Res..

[22] Gelardi M. (2026). Clinical-Cytological Grading in Chronic Rhinosinusitis with Nasal Polyps: An Integrated Framework for Precision Medicine. Cells.

[23] Staudacher A.G., Peters A.T., Kato A., Stevens W.W. (2020). Use of endotypes, phenotypes, and inflammatory markers to guide treatment decisions in chronic rhinosinusitis. Ann. Allergy Asthma Immunol..

[24] Bachert C., Marple B., Hosemann W., Cavaliere C., Wen W., Zhang N. (2020). Endotypes of chronic rhinosinusitis with nasal polyps: Pathology and possible therapeutic implications. J. Allergy Clin. Immunol. Pract..

[25] Paoletti G., Malvezzi L., Riccio A.M., Descalzi D., Pirola F., Russo E., De Ferrari L., Racca F., Ferri S., Messina M.R. (2022). Nasal cytology as a reliable non-invasive procedure to phenotype patients with type 2 chronic rhinosinusitis with nasal polyps. World Allergy Organ. J..

[26] Danisman Z., Linxweiler M., Kühn J.P., Linxweiler B., Solomayer E.F., Wagner M., Wagenpfeil G., Schick B., Berndt S. (2023). Differential nasal swab cytology represents a valuable tool for therapy monitoring but not prediction of therapy response in chronic rhinosinusitis with nasal polyps treated with dupilumab. Front. Immunol..

[27] Deutschle T., Friemel E., Starnecker K., Riechelmann H. (2005). Nasal cytologies: Impact of sampling method, repeated sampling and interobserver variability. Rhinology.

[28] Wang Y., Li Z., Lu J. (2024). Single-cell RNA sequencing reveals the epithelial cell, fibroblast, and key gene alterations in chronic rhinosinusitis with nasal polyps. Sci. Rep..

[29] Li J., Zhang X., Zhang Z., Song Y., Xu X., Zhang L., Zhang Y. (2026). AI-Based Quantitative Nasal Cytology for Predicting Eosinophilic Chronic Rhinosinusitis with Nasal Polyps: A Pilot Study. Int. Forum Allergy Rhinol..

[30] Zhang X., Xu X., Liu W., Qin L., Song Y., Li J., Xin L., Wang C., Zhang L., Zhang Y. (2026). Development of Artificial Intelligence for Quantitative Assessment of Nasal Inflammatory Cytology in Chronic Rhinitis by Whole-Slide Images. Int. Forum Allergy Rhinol..

